# Primary care characteristics and stage of cancer at diagnosis using data from the national cancer registration service, quality outcomes framework and general practice information

**DOI:** 10.1186/s12885-015-1497-1

**Published:** 2015-07-05

**Authors:** Rebecca Maclean, Mona Jeffreys, Alex Ives, Tim Jones, Julia Verne, Yoav Ben-Shlomo

**Affiliations:** 1 Speciality Registrar in Public Health, NHS England, South Plaza, Marlborough Street, Bristol, BS1 3NX UK; 2 Senior Lecturer in Epidemiology, School for Social and Community Medicine, Canynge Hall, 39 Whatley Road, Bristol, BS8 2PS UK; 3 Senior Analyst, Public Health England Knowledge and Intelligence team (South West), 1st floor, Grosvenor House, 149 Whiteladies Road, Bristol, BS8 2RA UK; 4 Research Assistant, NIHR CLAHRC West, 9th Floor, Whitefriars, Lewins Mead, Bristol, BS1 2NT UK; 5 Public Health England Knowledge and Intelligence team (South West), 1st floor, Grosvenor House, 149 Whiteladies Road, Bristol, BS8 2RA UK; 6 School for Social and Community Medicine, Canynge Hall, 39 Whatley Road, Bristol, BS8 2PS UK

**Keywords:** Delayed diagnosis, Neoplasms, General practice, Primary care, Quality indictors, health care

## Abstract

**Background:**

Survival from cancer is worse in England than in some European countries. To improve survival, strategies in England have focused on early presentation (reducing delay to improve stage at diagnosis), improving quality of care and ensuring equity throughout the patient pathway. We assessed whether primary care characteristics were associated with later stage cancer at diagnosis (stages 3/4 versus 1/2) for female breast, lung, colorectal and prostate cancer.

**Methods:**

Data obtained from the National Cancer Registration Service, Quality Outcomes Framework, GP survey and GP workforce census, linked by practice code. Risk differences (RD) were calculated by primary care characteristics using a generalised linear model, accounting for patient clustering within practices. Models were adjusted for age, sex and an area-based deprivation measure.

**Results:**

For female breast cancer, being with a practice with a higher two week wait (TWW) referral rate (RD −1.8 % (95 % CI −0.5 % to −3.2 %) p = 0.003) and a higher TWW detection rate (RD −1.7 % (95 % CI −0.3 % to −3.0 %) p = 0.003) was associated with a lower proportion diagnosed later. Being at a practice where people thought it less easy to book at appointment was associated with a higher percentage diagnosed later (RD 1.8 % (95 % CI 0.2 % to 3.4 %) p = 0.03). For lung cancer, being at practices with higher TWW referral rates was associated with lower proportion advanced (RD-3.6 % (95 % CI −1.8 %, −5.5 %) p < 0.001) whereas being at practices with more patients per GP was associated with higher proportion advanced (RD1.8 % (95 % CI 0.2, 3.4) p = 0.01). A higher rate of gastrointestinal investigations was associated with a lower proportion of later stage colorectal cancers (RD −2.0 % (95 % CI −0.6 % to −3.6 %) p = 0.01). No organisational characteristics were associated with prostate cancer stage.

**Conclusion:**

Easier access to primary care, faster referral and more investigation for gastrointestinal symptoms could reduce the proportion of people diagnosed later for female breast, lung and colorectal, but not prostate cancer. Differences between the four main cancers suggest different policies may be required for individual cancers to improve outcomes.

**Electronic supplementary material:**

The online version of this article (doi:10.1186/s12885-015-1497-1) contains supplementary material, which is available to authorized users.

## Background

Survival from cancer varies across European countries [1, 2]. Stage at diagnosis is strongly related to cancer mortality and more advanced stage at diagnosis may be associated with delay in diagnosis [3]. In England, The National Awareness and Early Diagnosis Initiative (NAEDI) was announced as part of the 2007 Cancer Strategy to understand and tackle reasons for more advanced stage at diagnosis in England compared to other EU countries [4]. To improve survival, strategies have focused on early presentation (reducing delay to improve stage at diagnosis), improving quality of care and ensuring equity throughout the patient pathway. Delays in diagnosis can be caused by delays in presentation, primary care delay (first presentation to referral), system delays (time to investigation) and secondary care delays (first seen in secondary care to diagnosis) [5, 6].

There has been little research investigating whether there is an association between characteristics and systems of primary care and stage of cancer at diagnosis. Research from Denmark showed associations between some primary care characteristics and patient or system delay [7]. The authors showed that patients attending a female doctor more often experienced short patient delay but longer system delay compared to patients attending a male doctor. Patients attending a practice with many services or seeing a doctor with little former knowledge of the patient more often experience short system delay. One recent study [8] found that higher total quality outcome framework (QOF) score protected against unplanned first-time admissions for cancer, but having no doctors with a UK primary medical qualification and being less able to offer appointments within 48 hrs were associated with increased odds of an unplanned first-time admission. Elliss-Brookes *et al.* [9] showed patients presenting via the emergency route have substantially lower 1-year relative survival than those presenting via other routes. Together, these studies indicate that primary care characteristics and systems could have an impact on cancer outcomes.

We investigated whether organisational characteristics of primary care practices in England were associated with stage at diagnosis of the four most common cancers (female breast, prostate, colorectal and lung cancer).

## Methods

### Data sources

Stage of cancer at diagnosis, patient-level demographic factors and primary care characteristics were obtained from a number of data sources.

#### Data linkage

**Fig. 1:**
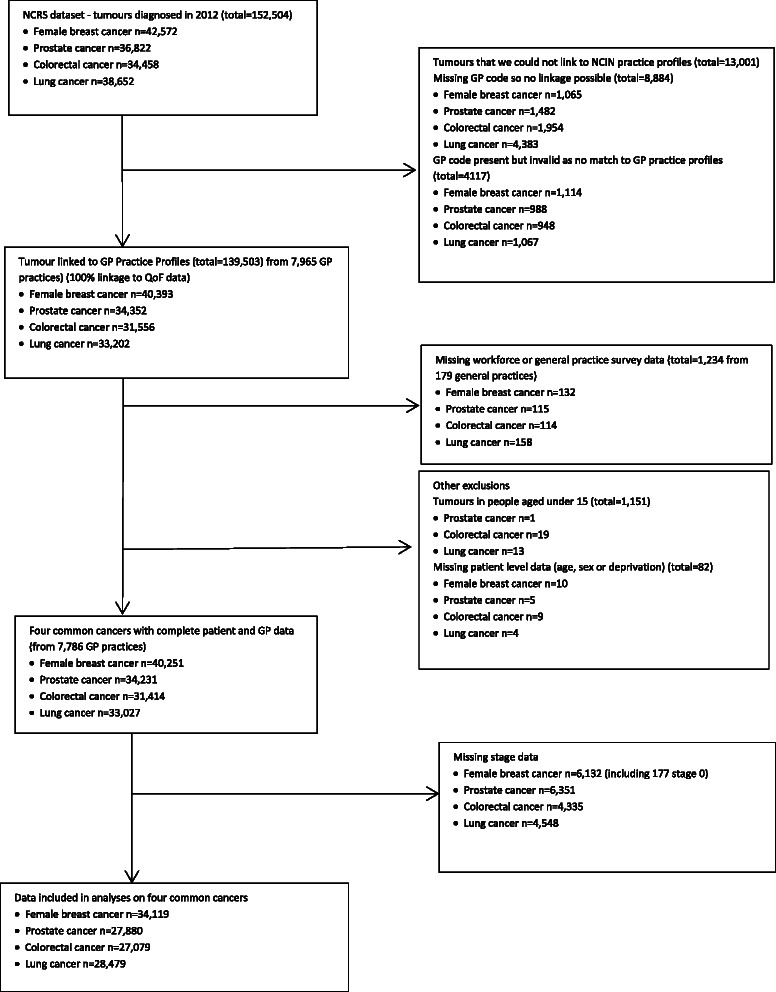
Data flow due to data linkage, missing data and exclusions from dataset

We were able to link across a numner of different datasets by using the unique GP code [10], where available and valid thereby providing us information on cancer characteristics, general practice level features and patient perceptions about their practice. This process and losses of data for a variety of different reasons including exclusions is shown in a flow diagram (Fig. 1)

*National Cancer Registration Service (NCRS)* [11]. There are eight offices of the NCRS in England which submit a standard dataset of information. Stage data was more than 70 % complete across England for female breast (ICD-10 C50), colorectal (ICD-10 C18 to C20), lung (ICD-10 C33 to C39, and C45) and prostate cancer (ICD-10 C61) [12]. We included stage data from all relevant fields within NCRS. (For a description of how stage data are collected within the NCRS see appendix 1 online). Data on patient age, sex, ethnicity and area-based deprivation (income-based domain of the index of multiple deprivation (IMD)) quintile were from NCRS dataset. NCRS information was provided by Public Health England’s National Cancer Registration Service; data from the cancer registry is publicly available but only once it has been aggregated to a level that is not patient-identifiable.

*National Cancer Intelligence Network (NCIN) Practice Profiles* [13]. These bring together data relevant to cancer in primary care from a range of sources. They were developed to provide information on general practice (GP) variation and understand cancer burden. Exposure variables from this data source were; two week wait (TWW) referral rate (number of TWW referrals for any cancer per 100,000 population), TWW conversion rate (percentage of all TWW referrals with cancer), TWW detection rate (percentage of new cancers treated which were referred through TWW system), average colonoscopy, sigmoidoscopy and endoscopy rate (average colonoscopy, sigmoidoscopy and upper gastrointestinal endoscopy in-patient or day case procedures, rate per 100,000), emergency admissions (number of persons admitted to hospital as an inpatient or day-case via an emergency admission, with a diagnostic code that includes cancer, per 100,000 population) and GP deprivation (income-based domain of IMD). Most data is freely available, however some small numbers within the profiles are only accessible through specific routes. A version of the GP Practice Profiles with potentially identifiable data suppressed is publicly available via the Public Health England National Cancer Intelligence Network’s Cancer Commissioning Toolkit.

*The Quality and Outcomes Framework (QOF)* is a financial incentive scheme that rewards GPs depending on their achievement against quality indicators [14]. The total QOF score was used with higher scores indicating better performance. The four domains within QOF (clinical, organisational, additional services and patient experience) were not used as separate variables as they were strongly correlated with each other and the total QOF score. The individual cancer indicator score was also strongly correlated with the total QOF score. Information on list size (number of patients per practice) was used with information on the number of general practitioners per practice (from the GP workforce census, see below) to calculate the average number of patients per general practitioner at each practice. QOF data is freely available, re-used with the permission of the Health and Social Care Information Centre.

*The General Practice Patient Survey* is a questionnaire sent to a random sample of adults registered at GPs across England [15]. It gives patients an opportunity to comment on their experience of their GP. Exposure variables from this data were; percentage of patients responding ‘yes’ to the question ‘Were you able to get an appointment see or speak to someone?’ 2011/12 and percentage of patients responding ‘always’, ‘almost always’ or ‘a lot of the time’ to the question ‘Were you able to see your preferred doctor?’ 2010/11. These aspects were chosen because studies have shown easier access (ability to get an appointment) and greater continuity (ability to see a preferred doctor) can be associated with reduced hospital admissions [16, 17]. In 2011/12, 2.74 million questionnaires were sent with a response rate of 38 % (5.56 million sent in 2010/11 with 36 % response rate). Data is freely available, re-used with the permission of the Health and Social Care Information Centre.

*General Practice workforce census* is collected annually and includes information on the numbers of general practitioners working in primary care [18]. Exposure variables from this data source were: age, gender and country of primary medical qualification of general practitioners, and the number of general practitioners per practice (full time equivalent). Single handed practice was not included as a separate exposure variable because there were only a small number (890, 11 %) of single handed practices. Data is freely available, re-used with the permission of the Health and Social Care Information Centre.

*Health & Social Care Information Centre (HSCIC) Indicator Portal* brings together health and social care indicators [19]. The rurality of GPs (based on population density of the GP postcode) was obtained from this source. Data is freely available, re-used with the permission of the Health and Social Care Information Centre.

(For more details and how we operationalised the exposure variables see the Additiona file 1: Table S’a’).

#### Inclusion/exclusion criteria

We included all practices that were in the NCIN Practice Profiles [13]. These were practices in the 2011/12 QOF data with the following exclusions; practices with a patient list size less than 1000, a greater than 10 % difference in list size between 2011/12 QOF and Attribution Dataset April 2010, practice was missing in Attribution Dataset April 2010 or the practice could not be allocated to a CCG. This resulted in 7,965 practices (158 of 8,123 practices within QOF 2011/12 were excluded).

### Statistical methods

Our primary outcome was the proportion of patients who were diagnosed with advanced cancer compared to those with an earlier stage. Our null hypothesis was that characteristics and systems of primary care would not influence the proportion with advanced versus earlier stage for each of our four specific cancer sites after accounting for patient-level demographic factors. We defined advanced stage as stages 3 or 4 (regional or metastatic) compared to stages 1 or 2 (locally confined) using data from the TNM classification (see appendix 1 for further description of staging).

We derived two sets of exposure variables (a) patient level (age, sex, ethnicity and area deprivation) and (b) primary care level. The latter were divided into four domains (i) GP demographics (ii) GP general performance (iii) GP specific cancer activities (iv) GP other activities.

We decided that we would use a risk difference rather than a risk ratio as the most appropriate effect estimate as this enables one to easily calculate the impact of a GP characteristic in absolute terms. We therefore used a generalised linear model for the binomial family with an identity link function. Our outcome variable, stage of cancer at diagnosis, was coded as zero for early stage (stages 1 or 2) and one for late stage (stages 3 or 4). We allowed errors in the model to be correlated within each GP practice to account for clustering of patients within GPs, thereby producing more conservative confidence inetrvals and p-values. Negative risk differences show that patients are less likely to be diagnosed at an advanced stage (3 or 4) compared to patients in the baseline group. The opposite is true for positive differences. Risk differences are presented as percentage risk difference. Analyses were conducted using STATA 13.

Female breast cancer and prostate cancer models were adjusted for age at diagnosis and patient level income-based deprivation. Colorectal and lung cancer models were adjusted for age at diagnosis, sex and patient level area-based deprivation. We developed a conceptual model (Additional file 1: Figure S’a’) on the potential inter-relationships between the primary care level factors. We had no *a priori* knowledge of this causal pathway and using the conceptual model decided not to mutually adjust for characteristics or systems of primary care as they may have been on the causal pathway and hence the coefficients from such a model would be misleading due to over-adjustment.

We undertook a series of sensitivity analyses to assess the impact of missing ethnicity data and of using stage data from different fields within NCRS. Missing data for stage of cancer at diagnosis was analysed to investigate whether there were systematic reasons for data being missing (missing not at random). Multiple imputation was used to generate missing values for stage for each of the four main cancers separately. The ice program was used to perform imputation in Stata 13. Imputation was performed on stage with sex, deprivation quintile and age included in the imputation model. A further model using the significant exposure variables for each cancer (female breast cancer included rurality, two week wait (TWW) referral rate, TWW detection rate, emergency admission rate, gender of general practitioners and ease of booking an appointment; prostate cancer included GP practice deprivation and practices rate of colonoscopy, sigmoidoscopy and endoscopy; colorectal cancer included practices rate of colonoscopy, sigmoidoscopy and endoscopy; lung cancer included TWW referral rate, TWW conversion rate, age and gender of general practitioners, number of patients per GP and emergency admission rates ). Twenty imputed data sets were created for each model.

## Results

There were 363,991 tumours diagnosed in 2012 (all cancers excluding non-melanoma skin cancers, ICD-10 C00 to C97 excluding C44). Of these there were 42,572 female breast cancers, 36,822 prostate cancers, 34,458 colorectal cancer and 38,652 lung cancers, accounting for 42 % of all cancers diagnosed in 2012. From these 34,119 female breast cancers (5,666 stage 3 or 4, 16.6 %), 27,880 prostate cancers (10,756 stage 3 or 4, 38.6 %), 27,079 colorectal cancers (14,793 stage 3 or 4, 54.6 %) and 28,479 lung cancers (21,520 stage 3 or 4, 75.6 %) were included in the analyses (see Fig. 1 for details of inclusion/exclusion of tumours). These were from patients at 7,786 GP practices across England.

(For details of the number of tumours of each cancer type by patient and GP variable see the Additional file 1: Table Sb).

At an individual level we found that various exposures could be important confounders for presenting with advanced female breast cancer (see Table 1). Non-white vs. white women and women living in more deprived areas were more likely to be diagnosed at a more advanced stage (RD 6.0 % (95 % CI 3.3 % to 8.6 %) p < 0.001; Q5 vs. Q1 RD 3.9 % (95 % CI 2.5 % to 5.3 %), p-value for trend <0.001).

**Table 1:** Univariate and adjusted risk differences for female breast cancer and prostate cancer

		Female breast cancer						Prostate cancer					
		Univariate			Adjusted; age & deprivation			Univariate			Adjusted; age & deprivation		
		Risk difference (95 % CI)		p-value	Risk difference (95 % CI)		p-value	Risk difference (95 % CI)		p-value	Risk difference (95 % CI)		p-value
Patient level	Age												
	65 + yrs	Baseline			Baseline			Baseline			Baseline		
	45-64 years	−3.2	(−4.1 to −2.4)	<0.001	−3.2	(−4.1 to −2.4)	<0.001	−8.1	(−9.4 to −6.8)	<0.001	−8.2	(−9.4 to −6.9)	<0.001
	15-44 years	2.1	(0.6 to 3.6)	0.01	1.9	(0.4 to 3.4)	0.01	−19.0	(−29.5 to −8.5)	<0.001	−19.7	(−30.2 to −9.3)	<0.001
	Ethnicity												
	White	Baseline						Baseline					
	Non-white	6.0	(3.3 to 8.6)	<0.001				−6.0	(−10.3 to −1.7)	0.01			
	Deprivation												
	Q1 (least deprived)	Baseline			Baseline			Baseline			Baseline		
	Q2	−0.6	(−1.8 to 0.6)		−0.8	(−1.9 to 0.4)		2.1	(0.5 to 3.8)		2.0	(0.4 to 3.7)	
	Q3	0.0	(−1.2 to 1.2)		−0.1	(−1.3 to 1.0)		2.7	(1.0 to 4.5)		2.6	(0.9 to 4.3)	
	Q4	2.5	(1.2 to 3.8)		2.2	(1.0 to 3.5)		4.1	(2.2 to 6.0)		4.2	(2.3 to 6.0)	
	Q5 (most deprived)	3.9	(2.5 to 5.3)	<0.001	3.6	(2.2 to 5.0)	<0.001	4.7	(2.7 to 6.8)	<0.001	4.9	(2.9 to 7.0)	<0.001
GP demographics	Number of patients per GP												
	Q1 (lowest)	Baseline			Baseline			Baseline			Baseline		
	Q2	−0.1	(−1.5 to 1.3)		−0.1	(−1.4 to 1.3)		−1.2	(−3.1 to 0.8)		−1.1	(−3.0 to 0.9)	
	Q3	−0.8	(−2.1 to 0.6)		−0.8	(−2.1 to 0.6)		−0.9	(−2.9 to 1.1)		−0.7	(−2.7 to 1.3)	
	Q4	0.3	(−1.1 to 1.7)		0.1	(−1.3 to 1.5)		−0.8	(−2.7 to 1.2)		−0.8	(−2.8 to 1.1)	
	Q5 (highest)	−0.1	(−1.5 to 1.2)	0.94	−0.6	(−1.9 to 0.7)	0.48	−2.3	(−4.2 to −0.4)	0.05	−2.3	(−4.2 to −0.4)	0.04
	Training practice												
	No	Baseline			Baseline			Baseline			Baseline		
	Yes	0.9	(0.1 to 1.8)	0.03	0.6	(−0.2 to 1.5)	0.16	−0.8	(−2.0 to 0.5)	0.23	−0.9	(−2.1 to 0.4)	0.18
	GPs aged 50 and over												
	Some	Baseline			Baseline			Baseline			Baseline		
	None	0.6	(−1.0 to 2.1)	0.46	0.3	(−1.2 to 1.8)	0.66	−0.5	(−2.7 to 1.8)	0.70	−0.5	(−2.8 to 1.7)	0.64
	All	1.5	(−0.4 to 3.3)	0.13	0.8	(−1.0 to 2.6)	0.41	−2.1	(−4.5 to 0.4)	0.10	−2.5	(−5.0 to −0.1)	0.04
	GPs female												
	Some	Baseline			Baseline			Baseline			Baseline		
	None	−0.1	(−1.7 to 1.6)	0.95	−0.7	(−2.3 to 0.9)	0.40	−0.5	(−2.7 to 1.7)	0.68	−1.0	(−3.1 to 1.2)	0.38
	All	5.0	(1.4 to 8.6)	0.01	4.0	(0.6 to 7.4)	0.02	−1.8	(−6.3 to 2.6)	0.42	−2.1	(−6.5 to 2.3)	0.34
	GPs qualified in UK												
	Some	Baseline			Baseline			Baseline			Baseline		
	None	1.5	(−0.4 to 3.4)	0.13	0.4	(−1.4 to 2.2)	0.68	−2.2	(−4.8 to 0.3)	0.08	−2.5	(−5.0 to 0.0)	0.05
	All	−0.4	(−1.3 to 0.5)	0.40	−0.3	(−1.1 to 0.6)	0.55	0.9	(−0.5 to 2.2)	0.20	1.1	(−0.2 to 2.4)	0.09
	GP level deprivation												
	Q1 (least deprived)	Baseline			Baseline			Baseline			Baseline		
	Q2	0.4	(−0.9 to 1.7)		0.3	(−1.0 to 1.5)		1.9	(0.1 to 3.7)		1.0	(−0.9 to 2.8)	
	Q3	0.1	(−1.2 to 1.4)		−0.6	(−1.9 to 0.7)		3.2	(1.3 to 5.0)		2.1	(0.2 to 4.0)	
	Q4	1.2	(−0.2 to 2.5)		−0.2	(−1.6 to 1.2)		3.5	(1.6 to 5.3)		2.1	(0.1 to 4.2)	
	Q5 (most deprived)	4.4	(2.9 to 5.9)	<0.001	2.5	(0.8 to 4.2)	0.14	3.5	(1.5 to 5.6)	<0.001	1.8	(−0.6 to 4.2)	0.04
	GP rurality												
	Urban > 10 K	Baseline			Baseline			Baseline			Baseline		
	Town and fringe	−2.4	(−3.5 to −1.4)	<0.001	−1.5	(−2.5 to −0.4)	0.01	−2.0	(−3.7 to −0.4)	0.01	−1.6	(−3.3 to 0.0)	0.05
	Village, hamlet & isolated dwellings	−2.5	(−4.7 to −0.4)	0.02	−1.6	(−3.7 to 0.4)	0.12	−2.0	(−5.1 to 1.0)	0.19	−1.2	(−4.3 to 1.9)	0.44
GP general performance	Able to book appointment												
	90 % and over	Baseline			Baseline			Baseline			Baseline		
	80-90 %	1.0	(0.1 to 1.9)		0.6	(−0.3 to 1.4)		0.9	(−0.4 to 2.2)		0.8	(−0.5 to 2.1)	
	<80 %	3.1	(1.5 to 4.7)	<0.001	1.7	(0.1 to 3.3)	0.04	−1.3	(−3.6 to 1.1)	0.92	−2.0	(−4.3 to 0.4)	0.70
	Able to see preferred GP												
	80 % and over	Baseline			Baseline			Baseline			Baseline		
	60-80 %	0.7	(−0.3 to 1.7)		0.4	(−0.6 to 1.4)		0.2	(−1.2 to 1.7)		0.2	(−1.2 to 1.6)	
	<60 %	1.7	(0.5 to 2.9)	0.01	1.0	(−0.2 to 2.2)	0.10	−0.7	(−2.5 to 1.0)	0.47	−0.9	(−2.7 to 0.9)	0.35
	Total QOF points												
	990 to 1000 (max) points	Baseline			Baseline			Baseline			Baseline		
	980 to 989 points	−0.2	(−1.3 to 0.9)		−0.4	(−1.4 to 0.7)		1.4	(−0.3 to 3.1)		1.3	(−0.4 to 2.9)	
	960 to 979 points	1.2	(0.0 to 2.4)		0.9	(−0.3 to 2.1)		1.2	(−0.5 to 2.8)		1.1	(−0.5 to 2.8)	
	<960 points	1.4	(0.0 to 2.7)	0.02	0.9	(−0.4 to 2.2)	0.11	0.7	(−1.3 to 2.6)	0.23	0.4	(−1.5 to 2.3)	0.75
GP specific cancer activities	Two week wait referral rate												
	Q1 (lowest)	Baseline			Baseline			Baseline			Baseline		
	Q2	−1.8	(−3.2 to −0.5)		−1.3	(−2.6 to 0.1)		0.1	(−1.8 to 1.9)		0.1	(−1.8 to 2.0)	
	Q3	−0.7	(−2.1 to 0.7)		−0.1	(−1.4 to 1.2)		1.6	(−0.3 to 3.6)		1.8	(−0.1 to 3.8)	
	Q4	−2.9	(−4.2 to −1.6)		−2.0	(−3.3 to −0.7)		1.4	(−0.5 to 3.4)		1.2	(−0.7 to 3.2)	
	Q5 (highest)	−2.3	(−3.6 to −0.9)	<0.001	−1.5	(−2.8 to −0.2)	0.01	0.7	(−1.2 to 2.7)	0.20	0.7	(−1.3 to 2.6)	0.26
	Two week wait conversion												
	Q1 (lowest)	Baseline			Baseline			Baseline			Baseline		
	Q2	−1.0	(−2.4 to 0.3)		−0.7	(−2.0 to 0.7)		2.1	(0.1 to 4.1)		2.0	(0.0 to 4.0)	
	Q3	−1.7	(−3.1 to −0.4)		−1.3	(−2.6 to 0.0)		1.1	(−0.9 to 3.1)		0.9	(−1.1 to 2.9)	
	Q4	−1.3	(−2.6 to 0.0)		−1.0	(−2.3 to 0.3)		0.9	(−1.1 to 2.9)		0.7	(−1.3 to 2.7)	
	Q5 (highest)	−1.0	(−2.4 to 0.3)	0.12	−0.7	(−2.0 to 0.6)	0.23	1.6	(−0.3 to 3.5)	0.34	1.4	(−0.5 to 3.3)	0.46
	Two week wait detection												
	Q1 (lowest)	Baseline			Baseline			Baseline			Baseline		
	Q2	−0.5	(−1.9 to 0.8)		−0.2	(−1.6 to 1.1)		0.5	(−1.4 to 2.4)		0.5	(−1.5 to 2.4)	
	Q3	−1.6	(−2.9 to −0.2)		−1.1	(−2.4 to 0.2)		1.9	(0.0 to 3.8)		1.9	(0.0 to 3.8)	
	Q4	−2.6	(−4.1 to −1.2)		−1.9	(−3.3 to −0.6)		2.8	(0.7 to 4.8)		2.7	(0.7 to 4.8)	
	Q5 (highest)	−2.0	(−3.4 to −0.6)	<0.001	−1.3	(−2.6 to 0.0)	0.01	0.3	(−1.7 to 2.2)	0.26	0.6	(−1.3 to 2.5)	0.15
GP other activities	Average colonoscopy, sigmoidoscopy and upper GI endoscopy												
	T1 (lowest)	Baseline			Baseline			Baseline			Baseline		
	T2	−0.8	(−1.9 to 0.2)		−0.6	(−1.6 to 0.4)		2.3	(0.8 to 3.8)		2.4	(0.9 to 3.9)	
	T3 (highest)	0.5	(−0.5 to 1.6)	0.33	0.6	(−0.4 to 1.6)	0.28	2.5	(1.0 to 4.0)	0.001	2.4	(0.9 to 3.9)	0.002
	Emergency admissions												
	Q1 (lowest)	Baseline			Baseline			Baseline			Baseline		
	Q2	−1.9	(−3.2 to −0.5)		−1.6	(−2.8 to −0.3)		1.2	(−0.7 to 3.2)		1.5	(−0.4 to 3.4)	
	Q3	−0.3	(−1.7 to 1.0)		−0.1	(−1.5 to 1.2)		1.7	(−0.2 to 3.6)		1.7	(−0.2 to 3.6)	
	Q4	−1.0	(−2.3 to 0.3)		−0.8	(−2.1 to 0.5)		1.7	(−0.3 to 3.6)		1.4	(−0.6 to 3.3)	
	Q5 (highest)	−2.0	(−3.4 to −0.7)	0.04	−2.0	(−3.3 to −0.8)	0.03	2.1	(0.1 to 4.0)	0.04	1.6	(−0.4 to 3.5)	0.17

Women aged 15–44 years were more likely to be diagnosed at a more advanced stage than women aged 65 years and over whereas women aged 45–64 years were less likely to be diagnosed at a more advanced stage (15-44years vs. 65+ RD 2.1 % (95 % CI 0.6 % to 3.6 %) p = 0.01; 45–64 years vs. 65+ RD −3.2 % (95 % CI −4.1 % to −2.4 %) p < 0.001).

A variety of GP exposures were associated with stage at presentation but after adjustment for age and deprivation the following predicted lower proportion with advanced stage female breast cancer: having a GP in a town/fringe area compared to urban area (RD −1.5 % (95 % CI −2.5 % to −0.4 %) p = 0.01), ), practices with higher two week wait (TWW) referral rate and a higher TWW detection rate (Q5 vs. Q1 RD −1.5 % (95 % CI −2.8 % to −0.2 %) p value for trend = 0.01; Q5 vs. Q1 RD −1.3 % (95 % CI −2.6 % to 0.0 %) p value for trend = 0.01) and practices that had a higher emergency admission rate (Q5 vs. Q1 RD −2.0 % (95 % CI −3.3 % to −0.8 %) p value for trend = 0.03). In contrast having only female general practitioners at the practice and being at a practice where people thought it was less easy to book an appointment was associated with a higher percentage diagnosed at a more advanced stage (all female GPs: RD 4.0 % (95 % CI 0.6 % to 7.4 %) p = 0.02; <80 % thought easy to book appointment compared to >90 % RD 1.7 % (95 % CI 0.1 % to 3.3 %) p = 0.04.

At the individual level we found that various exposures could be important confounders for presenting with advanced prostate cancer (see table 1). Men living in more deprived areas were more likely to be diagnosed at a more advanced stage than those living in less deprived areas (Q5 vs. Q1 RD 4.7 % (95 % CI 2.7 % to 6.8 %), p-value for trend <0.001). Non-white vs. white men and younger men were less likely to be diagnosed at a more advanced stage (RD −6.0 % (95 % CI −10.3 % to −1.7 %) p = 0.01; 45-64 years vs. 65+ RD −8.1 % (95 % CI −9.4 % to −6.8 %) p < 0.001, 15-44 years vs. 65+ RD −19.0 % (95 % CI −29.5 % to −8.5 %) p < 0.001).

After adjustment for age and deprivation GP practice deprivation and practices with higher rates of colonoscopy, sigmoidoscopy and endoscopy were associated with a higher percentage diagnosed at a more advanced stage (Q5 vs. Q1 RD 1.8 % (95 % CI −0.6 % to 4.2 %) p-value for trend 0.04; tertile 3 vs. tertile 1 RD 2.4 % (95 % CI 0.9 % to 3.9 %) p value for trend = 0.002).

For colorectal cancer, at the individual level, we found that various exposures could be important confounders for presenting later (see Table 2). Non-white vs. white people and younger people were more likely to be diagnosed at a more advanced stage (RD 6.7 % (95 % CI 2.7 % to 10.7 %) p = 0.001; 15-44 years vs. 65+ RD 10.3 % (95 % CI 7.1 % to 13.4 %) p < 0.001, 45-64 years vs. 65+ RD 6.0 % (95 % CI 4.6 % to 7.3 %) p < 0.001). After adjustment for age, sex and deprivation the only GP exposure which was associated with stage at presentation was the average colonoscopy, sigmoidoscopy and endoscopy rate. We found that a higher average colonoscopy, sigmoidoscopy and endoscopy rate was associated with a lower percentage of people diagnosed at a more advanced stage (tertile 3 vs. tertile 1 RD −2.0 % (95%CI −3.5 % to −0.5 %) p value for trend = 0.01).

**Table 2:** Univariate and adjusted risk differences for colorectal cancer and lung cancer

		Colorectal cancer						Lung cancer					
		Univariate			Adjusted; age & deprivation			Univariate			Adjusted; age & deprivation		
		Risk difference (95 % CI)		p-value	Risk difference (95 % CI)		p-value	Risk difference (95 % CI)		p-value	Risk difference (95 % CI)		p-value
Patient level	Age												
	65 + yrs	Baseline			Baseline			Baseline			Baseline		
	45-64 years	6.0	(4.6 to 7.3)	<0.001	5.9	(4.6 to 7.3)	<0.001	3.1	(1.7 to 4.5)	<0.001	3.3	(1.9 to 4.6)	<0.001
	15-44 years	10.3	(7.1 to 13.4)	<0.001	10.1	(6.9 to 13.3)	<0.001	4.2	(−1.5 to 9.9)	0.15	4.5	(−1.2 to 10.2)	0.12
	Sex												
	Male	Baseline			Baseline			Baseline			Baseline		
	Female	0.1	(−1.1 to 1.4)	0.82	0.0	(−1.2 to 1.2)	1.00	−3.1	(−4.1 to −2.1)	<0.001	−3.3	(−4.3 to −2.3)	<0.001
	Ethnicity												
	White	Baseline						Baseline					
	Non-white	6.7	(2.7 to 10.7)	0.001				−0.7	(−4.6 to 3.1)	0.71			
	Deprivation												
	Q1 (least deprived)	Baseline			Baseline			Baseline			Baseline		
	Q2	−0.4	(−2.2 to 1.4)		−0.3	(−2.1 to 1.5)		−0.5	(−2.2 to 1.3)		−0.4	(−2.2 to 1.4)	
	Q3	−0.3	(−2.2 to 1.5)		−0.3	(−2.1 to 1.6)		0.3	(−1.4 to 2.1)		0.4	(−1.3 to 2.1)	
	Q4	1.0	(−0.9 to 2.9)		0.9	(−1.0 to 2.8)		−0.3	(−2.0 to 1.4)		−0.4	(−2.1 to 1.3)	
	Q5 (most deprived)	1.5	(−0.6 to 3.5)	0.07	1.1	(−0.9 to 3.1)	0.14	−1.0	(−2.7 to 0.7)	0.29	−1.3	(−3.0 to 0.4)	0.13
GP demographics	Number of patients per GP												
	Q1 (lowest)	Baseline			Baseline			Baseline			Baseline		
	Q2	1.0	(−0.9 to 2.9)		1.1	(−0.8 to 3.0)		0.5	(−1.1 to 2.2)		0.6	(−1.0 to 2.2)	
	Q3	1.1	(−0.9 to 3.1)		1.1	(−0.9 to 3.1)		0.6	(−1.0 to 2.3)		0.7	(−0.9 to 2.3)	
	Q4	1.6	(−0.3 to 3.6)		1.5	(−0.4 to 3.4)		1.7	(0.1 to 3.3)		1.7	(0.2 to 3.3)	
	Q5 (highest)	0.4	(−1.6 to 2.4)	0.54	0.2	(−1.8 to 2.1)	0.74	2.0	(0.4 to 3.5)	0.01	1.8	(0.2 to 3.4)	0.01
	Training practice												
	No	Baseline			Baseline			Baseline			Baseline		
	Yes	0.2	(−1.0 to 1.5)	0.71	0.0	(−1.2 to 1.3)	0.95	0.6	(−0.5 to 1.6)	0.27	0.6	(−0.5 to 1.6)	0.28
	GPs aged 50 and over												
	Some	Baseline			Baseline			Baseline			Baseline		
	None	−1.2	(−3.5 to 1.1)	0.30	−1.4	(−3.6 to 0.9)	0.24	−2.6	(−4.3 to −0.8)	0.01	−2.5	(−4.3 to −0.7)	0.01
	All	0.9	(−1.7 to 3.4)	0.52	0.5	(−2.1 to 3.1)	0.69	2.0	(−0.1 to 4.1)	0.07	2.0	(−0.1 to 4.1)	0.06
	GPs female												
	Some	Baseline			Baseline			Baseline			Baseline		
	None	−0.4	(−2.7 to 1.9)	0.73	−0.8	(−3.1 to 1.6)	0.53	1.3	(−0.5 to 3.1)	0.17	1.3	(−0.5 to 3.1)	0.14
	All	−3.0	(−8.2 to 2.2)	0.27	−3.5	(−8.7 to 1.7)	0.19	−4.5	(−8.4 to −0.6)	0.03	−4.6	(−8.4 to −0.7)	0.02
	GPs qualified in UK												
	Some	Baseline			Baseline			Baseline			Baseline		
	None	−0.1	(−2.7 to 2.6)	0.95	−0.5	(−3.2 to 2.2)	0.71	−0.6	(−2.6 to 1.5)	0.59	−0.5	(−2.6 to 1.5)	0.61
	All	−0.6	(−2.0 to 0.7)	0.35	−0.4	(−1.8 to 0.9)	0.51	−0.2	(−1.3 to 0.9)	0.68	−0.3	(−1.4 to 0.8)	0.61
	GP level deprivation												
	Q1 (least deprived)	Baseline			Baseline			Baseline			Baseline		
	Q2	−1.2	(−3.1 to 0.7)		−1.4	(−3.3 to 0.6)		−1.8	(−3.5 to −0.1)		−1.8	(−3.5 to −0.1)	
	Q3	−1.0	(−2.9 to 0.8)		−1.3	(−3.3 to 0.7)		−0.3	(−2.0 to 1.4)		−0.6	(−2.3 to 1.2)	
	Q4	0.5	(−1.4 to 2.5)		−0.2	(−2.4 to 1.9)		−0.9	(−2.5 to 0.8)		−1.2	(−3.0 to 0.7)	
	Q5 (most deprived)	0.9	(−1.2 to 3.0)	0.17	−0.4	(−2.9 to 2.1)	1.00	−2.6	(−4.3 to −0.9)	0.03	−2.8	(−4.8 to −0.8)	0.04
	GP rurality												
	Urban > 10 K	Baseline			Baseline			Baseline			Baseline		
	Town and fringe	−0.7	(−2.5 to 1.0)	0.40	−0.1	(−1.9 to 1.6)	0.87	0.0	(−1.5 to 1.5)	0.96	−0.2	(−1.7 to 1.4)	0.83
	Village, hamlet & isolated dwellings	−0.3	(−3.5 to 2.8)	0.84	0.2	(−2.9 to 3.3)	0.90	0.7	(−2.5 to 3.9)	0.67	0.4	(−2.9 to 3.6)	0.82
GP general performance	Able to book appointment												
	90 % and over	Baseline			Baseline			Baseline			Baseline		
	80-90 %	0.1	(−1.2 to 1.4)		−0.1	(−1.4 to 1.2)		−0.7	(−1.7 to 0.4)		−0.5	(−1.6 to 0.6)	
	<80 %	1.1	(−1.3 to 3.5)	0.46	0.3	(−2.1 to 2.7)	0.95	−0.5	(−2.4 to 1.4)	0.32	−0.3	(−2.2 to 1.7)	0.52
	Able to see preferred GP												
	80 % and over	Baseline			Baseline			Baseline			Baseline		
	60-80 %	−0.6	(−2.0 to 0.9)		−0.6	(−2.1 to 0.8)		−1.4	(−2.6 to −0.2)		−1.3	(−2.5 to 0.0)	
	<60 %	0.5	(−1.3 to 2.3)	0.65	0.1	(−1.7 to 1.9)	0.96	−1.4	(−2.8 to 0.0)	0.05	−1.2	(−2.6 to 0.2)	0.09
	Total QOF points												
	990 to 1000 (max) points	Baseline			Baseline			Baseline			Baseline		
	980 to 989 points	0.1	(−1.4 to 1.7)		0.0	(−1.6 to 1.5)		−0.8	(−2.1 to 0.5)		−0.7	(−2.0 to 0.6)	
	960 to 979 points	0.6	(−1.2 to 2.4)		0.4	(−1.4 to 2.2)		−0.4	(−1.8 to 1.0)		−0.5	(−1.9 to 0.9)	
	<960 points	−0.5	(−2.5 to 1.4)	0.93	−0.9	(−2.8 to 1.1)	0.53	−0.8	(−2.4 to 0.8)	0.29	−0.7	(−2.3 to 0.9)	0.65
GP specific cancer activities	Two week wait referral rate												
	Q1 (lowest)	Baseline			Baseline			Baseline			Baseline		
	Q2	0.3	(−1.6 to 2.3)		0.6	(−1.3 to 2.5)		−1.6	(−3.2 to −0.1)		−1.6	(−3.2 to −0.1)	
	Q3	−0.6	(−2.5 to 1.3)		−0.3	(−2.2 to 1.7)		−2.3	(−3.8 to −0.7)		−2.3	(−3.9 to −0.8)	
	Q4	−0.7	(−2.6 to 1.3)		−0.2	(−2.1 to 1.8)		−2.1	(−3.7 to −0.5)		−2.0	(−3.6 to −0.5)	
	Q5 (highest)	−1.2	(−3.1 to 0.8)	0.13	−0.6	(−2.6 to 1.4)	0.39	−3.4	(−5.0 to −1.8)	<0.001	−3.3	(−4.9 to −1.7)	<0.001
	Two week wait conversion												
	Q1 (lowest)	Baseline			Baseline			Baseline			Baseline		
	Q2	0.8	(−1.2 to 2.7)		1.0	(−1.0 to 3.0)		1.3	(−0.3 to 3.0)		1.5	(−0.1 to 3.1)	
	Q3	−2.5	(−4.5 to −0.5)		−2.0	(−4.0 to −0.1)		1.2	(−0.4 to 2.9)		1.3	(−0.4 to 2.9)	
	Q4	−1.8	(−3.7 to 0.2)		−1.3	(−3.2 to 0.7)		3.6	(2.0 to 5.2)		3.6	(2.0 to 5.2)	
	Q5 (highest)	0.6	(−1.3 to 2.6)	0.56	1.2	(−0.7 to 3.1)	0.96	4.1	(2.5 to 5.7)	<0.001	4.0	(2.4 to 5.6)	<0.001
	Two week wait detection												
	Q1 (lowest)	Baseline			Baseline			Baseline			Baseline		
	Q2	1.5	(−0.5 to 3.4)		1.7	(−0.2 to 3.7)		−1.1	(−2.7 to 0.5)		−1.1	(−2.6 to 0.5)	
	Q3	0.7	(−1.2 to 2.6)		0.9	(−0.9 to 2.8)		−1.0	(−2.5 to 0.6)		−0.8	(−2.4 to 0.7)	
	Q4	−1.2	(−3.3 to 0.9)		−0.8	(−2.9 to 1.2)		−0.7	(−2.3 to 0.9)		−0.6	(−2.2 to 1.1)	
	Q5 (highest)	1.3	(−0.6 to 3.3)	0.92	1.6	(−0.4 to 3.5)	0.72	−1.3	(−2.9 to 0.3)	0.22	−1.3	(−2.9 to 0.3)	0.23
GP other activities	Average colonoscopy, sigmoidoscopy and upper GI endoscopy												
	T1 (lowest)	Baseline			Baseline			Baseline			Baseline		
	T2	−0.3	(−1.8 to 1.2)		0.0	(−1.5 to 1.5)		−1.1	(−2.4 to 0.1)		−1.1	(−2.3 to 0.2)	
	T3 (highest)	−2.4	(−3.9 to −0.9)	0.002	−2.0	(−3.5 to −0.5)	0.01	−0.7	(−2.0 to 0.5)	0.27	−0.5	(−1.7 to 0.8)	0.48
	Emergency admissions												
	Q1 (lowest)	Baseline			Baseline			Baseline			Baseline		
	Q2	−0.2	(−2.1 to 1.8)		0.2	(−1.8 to 2.1)		0.4	(−1.3 to 2.0)		0.4	(−1.2 to 2.1)	
	Q3	−0.7	(−2.6 to 1.3)		−0.3	(−2.2 to 1.7)		1.0	(−0.6 to 2.7)		1.0	(−0.6 to 2.7)	
	Q4	−0.7	(−2.7 to 1.3)		−0.2	(−2.2 to 1.7)		0.8	(−0.8 to 2.3)		0.9	(−0.7 to 2.5)	
	Q5 (highest)	−0.9	(−2.8 to 1.1)	0.32	−0.3	(−2.2 to 1.7)	0.66	1.3	(−0.3 to 2.9)	0.10	1.6	(0.0 to 3.2)	0.04

Age and gender were important confounders for presenting with advanced lung cancer (see Table 2). Women were less likely to be diagnosed at a more advanced stage than men (RD −3.3 % (95 % CI-4.3 % to −2.3 %) p < 0.001). People aged 45–64 years were more likely to be diagnosed at a more advanced stage than people aged 65 and over (RD 3.3 % (95 % CI 1.9 % to 4.6 %) p < 0.001) but there was no difference between people aged 15–44 years and people 65 and over (RD 4.5 % (95 % CI −1.2 % to 10.2 %) p = 0.12).

After adjustment for age, sex and deprivation, being at a practice with a higher TWW referral rate, having no GPs aged 50 and over and having all female GPs was associated with a lower percentage diagnosed with more advanced stage lung cancer (Q5 vs. Q1 RD-3.3 % (95 % CI −4.9 % to −1.7 %) p-value for trend <0.001; none vs. some RD-2.5 % (95%CI −4.3 % to −0.7 %) p = 0.01; all vs some. RD-4.6 % (95%CI −8.4 % to −0.7 %) p = 0.02). In contrast being at a practice which had more patients per GP, being at a practice with a higher TWW conversion rate and being at a practice that had a higher emergency admission rate was associated with a higher percentage diagnosed at a more advanced stage (Q5 vs. Q1 RD 1.8 % (95 % CI0.2 % to 3.4 %), p-value for trend 0.01; Q5 vs. Q1 RD 4.0 % (2.4 % to 5.5 %) p-value for trend <0.001; Q5 vs. Q1 RD 1.6 % (95%CI 0.0 % to 3.2 %) p-value fpr trend 0.04). There is a weak negative correlation between TWW referral and TWW conversion and this may explain some of the association between higher TWW conversion and more advanced stage at diagnosis.

### Missing stage data and multiple imputation

There was no systematic pattern of missing stage data between patient age and sex across the four common cancers. For female breast, prostate and lung cancer people who were more deprived were less likely to have missing stage data. Comparison of risk difference with and without the use of stage imputation shows very small alterations to risk differences which did not alter trends or interpretation for exposure variables.

### Sensitivity analysis

For cancers with stage data ethnicity was missing for 36.1 % of patients with female breast cancer, 47.9 % of prostate cancer, 33.1 % of colorectal cancer and 30.7 % of lung cancer. To assess the impact of adjusting for ethnicity, results for patients with complete ethnicity data adjusted for age, sex, deprivation and ethnicity were compared to an analysis excluding ethnicity. There were only very small changes in risk differences between these analyses with no change to the trends or conclusions drawn from the results. This is probably due to the distribution of ethnicity with 96 % of those with staged female breast, colorectal, lung and prostate cancer being white.

The main analysis used all relevant stage data from NCRS (see Additional file 1: appendix 1 for description of collection of stage data). If only the data from the NCRS ‘Stage best’ field was used 32,590 (81.0 %) of female breast cancers had staging data, 26,847 (78.4 %) of prostate cancers, 25,362 (80.7 %) of colorectal cancer and 27,134 (82.2 %) of lung cancers. Analysis to assess the impact of using all relevant stage data compared to using the ‘Stage Best’ field alone showed very small changes to the risk differences for female breast, colorectal and lung cancer. There was no change to the trends or conclusions of the results. For prostate cancer there were some slightly greater changes to the risk differences.

Due to the large proportion of lung cancers diagnosed at stage 3 or 4 we conducted an analysis to compare stage 4 with stage 1, 2 or 3. The trends for number of patients per GP, TWW referral rate and TWW conversion rate did not alter. However the relationship between GP demographics (age and gender) and emergency admissions were attenduated.

## Discussion

We have observed that some characteristics and systems of primary care practices are associated with the stage of cancer at diagnosis, but these vary by cancer type. If these associations are causal, then these results have important policy implications and could reduce cancer mortality rates for these four cancers.

For female breast cancer being at a practice where people thought it was easier to get an appointment and being at a practice more likely to use the TWW referral system may reduce more advanced stage at diagnosis. Having only female general practitioners may hinder diagnosis at an earlier stage. This reflects findings by Hansen *et al.* [7] that even though patients of female doctors had shorter patient delays they more often experienced longer system delays. These may suggest that access to primary care and speed of referral to secondary care are important in the earlier diagnosis of female breast cancer.

For prostate cancer the picture is more mixed with individual characteristics having a large influence on stage at diagnosis which may suggest differences are due to underlying tumour biology and factors affecting patient delay. Counter-intuitively, higher rates of colonoscopy, sigmoidoscopy and endoscopy were associated with more advanced stage at diagnosis. It is possible this reflects a higher prevalence of gastrointestinal symptoms in areas where less prostate specific antigen (PSA) testing is being done, some practices focus more on colorectal cancer than prostate cancer, or this was a type I error.

Being at a practice using more investigations for gastrointestinal symptoms appeared to reduce more advanced stage diagnosis of colorectal cancer. This echoes research which showed that screening sigmoidoscopy and colonoscopy reduced colorectal cancer mortality [20]. Younger patients were more likely to present with more advanced cancers as has been noted previously in the literature [21].

For lung cancer having fewer patients per general practitioner, being at a practice more likely to use the TWW referral system and at a practice where a larger proportion of cancers are diagnosed through TWW may reduce more advanced stage diagnosis. This could suggest that access to primary care and speed of referral to secondary care could be important in the early diagnosis of lung cancer. Interestingly men were more likely to present with advanced cancers, which could reflect health care seeking behaviours but this pattern was not seen for colorectal cancer. Alternatively it may reflect different smoking behaviour as male smokers consume more cigarettes per day than women [22]. For both breast and prostate cancers, practices in urban areas did less well than those in towns and this may reflect the greater burden of primary care work in such areas despite our attempts to adjust for patient level deprivation.

Hansen *et al.* [7] found that in Denmark, GP characteristics such as perceived GP accessibility and opportunities for referring were associated with patient and system delay. This is similar to our findings that access to GP (number of patients per GP and perceived ease of getting an appointment) and use of TWW were associated with reduced proportion of patients diagnosed at a more advanced stage for breast and lung cancer. We found no evidence of an association between being able to see a preferred GP and stage of cancer at diagnosis which differ from findings by Rogers *et al.* [23] who showed a negative association between seeing a preferred GP and cancer detection rate. We found no evidence of an association between stage and total QOF points which reflects similar findings by Levene *et al.* [24] with regards to specific QOF indicators and cancer mortality. However this is different to the findings of Bottle *et al.* [8] who found that higher QOF protected against unplanned first-time admissions for cancer. This may suggest that QOF score is important in certain aspects of the patient journey. We found no evidence that people registered at rural GP practices were more likely to be diagnosed at a more advanced stage than those living in urban areas. In relation to patient level differences our findings are similar to other studies [25]. However our finding in relation to age and stage of breast cancer are slightly unusual but this may be the result of including cases diagnosed clinically with those diagnosed by screening.

We have analysed data from a large proportion of four of the most common cancers diagnosed in 2012. Linking this to routinely collected data allowed us to analyse a wide range of characteristics of primary care. Due to the large number of exposure variables we conducted multiple testing however where the p-value is very small chance findings remain unlikely. It is worth noting where risk differences are very small that even though statistically significant this may be due to the large sample size. We have focused on primary care as an important aspect in diagnostic delay but there were some aspects we could not include, for example general practitioner related factors such as communication skills and trust, differences between general practitioners within GP and number of consultations at GPs [6, 23, 26, 27]. We also could not account for many patient factors (psychosocial factors, emotional response, support, co-morbidities or individual hospital use) or secondary care factors (different oncology services and radiological investigations) [9, 28–30]. TWW referral rate may be influenced by primary and secondary care aseven though primary care makes the referrals if these are not seen within 2 weeks they do not count as TWW. Further limitations include the high percentage of missing ethnicity data which meant we were unable to include this in the multivariable models. We could not distinguish women with breast cancer diagnosed by screening rather than symptomatic presentation as these data were incomplete within the NCRS. This could alter the implications of the findings if there was a correlation between exposure variables and screening detection rates at practices. In addition there is little variation in the number of days primary care delay for breast cancer [31] once patients have presented. However characteristics of primary care could still influence whether patients delay in seeking care in the first place. We could also not distinguish people with colorectal cancer diagnosed by screening rather than symptomatic presentation. This could be important if organizational or patient level factors influencing the effectiveness of the screening programme are themselves correlated with GP level factors, which may or may not be true.

More advanced stage was used as a proxy marker for a poor outcome since more advanced stage is related to lower survival. By diagnosing someone earlier (stage 3 to stage 2 or stage 4 to stage 3 for lung cancer) one year relative survival improves; female breast cancer 91 % to 98 %, prostate cancer 99 % to 100 %, colorectal cancer 87 % to 91 % and lung cancer 15 % to 36 % [32]. Given the large number of people diagnosed with breast, colorectal, lung and prostate cancer even small risk differences have the potential to make large differences to survival. Improving access to primary care and use of TWW may reduce more advanced stage at diagnosis for breast and lung cancer, and therefore improve survival. Use of investigations for gastrointestinal symptoms could be important to reduce more advanced stage at diagnosis, though one must also consider the impact of inappropriate investigations and the cost of these procedures.

## Conclusion

We have shown that higher use of TWW may reduce more advanced stage at diagnosis. The varied use and impact of TWW referral rate, conversion rate and detection rate along with controversy relating to the TWW criteria highlight this as a potential area for further research [33, 34]. In addition further research is required to understand how and in what circumstances TWW is most effective and cost-effective, integrating risk assessment tools into this policy [35]. Our results suggest that improving access to primary care, efficient use of the referral systems and faster investigations may reduce more advanced stage diagnosis for female breast cancer, colorectal cancer and lung cancer. However which apects of these areas and the exact way that they may reduce advanced stage at diagnosis requires further understanding. There were differences between the four main cancers which suggest different policies may be required for individual cancers to improve outcomes.

### Ethics

Ethics review was not required for this study.

## Additional file

Additional file 1: **Appendix 1 – national cancer registration service (ncrs) data.** Supplementary table ‘a’ – exposure variables explained. Supplementary figure ‘a’ – conceptual model. Supplementary table ‘b’ – number and percentage of tumours of each cancer type and stage.
