# Supplementary material for: Primary care characteristics and stage of cancer at diagnosis using data from the national cancer registration service, quality outcomes framework and general practice information
Source: BMC Cancer. 2015 Jul 5;15:500. doi: 10.1186/s12885-015-1497-1 (PMC4491217; doi:10.1186/s12885-015-1497-1)
Supplement: Additional file 1: — Appendix 1 – national cancer registration service (ncrs) data. Supplementary table ‘a’ – exposure variables explained. Supplementary figure ‘a’ – conceptual model. Supplementary table ‘b’ – number and percentage of tumours of each cancer type and stage. [file 12885_2015_1497_MOESM1_ESM.pdf]

APPENDIX 1 – NATIONAL CANCER REGISTRATION SERVICE (NCRS) DATA

The NCRS for England follows the Union for International Cancer Control (UICC) 'TNM' tumour staging criteria. Some registry offices enter staging information as a number in the 'Stage Best' field in the NCRS. Other registry offices enter staging information in the 'T Best', 'N Best', and 'M Best' fields and/or the 'T Path', 'N Path', and 'M Path' fields (which are based on pathology). Some registry offices enter information in both. Where there was a valid entry in the 'Stage Best' field, this was used; otherwise stage was derived from the separate 'T', 'N', and 'M' fields by applying the UICC TNM staging criteria (version 7) to the 'Best' fields if they contained information and the 'Path' fields if not.

<u>TNM classification for each cancer site</u>
Source: Sobin LH, Gospodarowicz MK, Wittekind C. TNM Classification of Malignant Tumours, 7th edition. John Wiley & Sons, 2011.

Breast Cancer:

	**Stage**	**T**	**N**	**M**
Stage 1	I	1	0	0
Stage 2	Iia	0	1	0
Stage 2	IIa	1	1	0
Stage 2	IIa	2	0	0
Stage 2	IIb	2	1	0
Stage 2	IIb	3	0	0
Stage 3	IIIa	0	2	0
Stage 3	IIIa	1	2	0
Stage 3	IIIa	2	2	0
Stage 3	IIIa	3	1 or 2	0
Stage 3	IIIb	4	Any	0
Stage 3	IIIb	Any	3	0
Stage 4	IV	Any	Any	1

Colorectal Cancer:

	**Stage**	**T**	**N**	**M**
Stage 1	I	1	0	0
Stage 1	I	2	0	0
Stage 2	II	3	0	0
Stage 2	II	4	0	0
Stage 3	III	Any	1 or 2	0
Stage 4	IV	Any	Any	1

Lung Cancer:

	Stage	T	N	M
Stage 1	Ia	1	0	0
Stage 1	Ib	2	0	0
Stage 2	IIa	1	1	0
Stage 2	IIb	2	1	0
Stage 2	IIb	3	0	0
Stage 3	IIIa	1	2	0
Stage 3	IIIa	2	2	0
Stage 3	IIIa	3	1 or 2	0
Stage 3	IIIb	Any	3	0
Stage 3	IIIb	4	Any	0
Stage 4	IV	Any	Any	1

Prostate Cancer:

	Stage	T	N	M	Grade
Stage 1	I	1a	0	0	G1
Stage 2	II	1a	0	0	G2-4
Stage 2	II	1b	0	0	Any
Stage 2	II	1c	0	0	Any
Stage 2	II	1	0	0	Any
Stage 2	II	2	0	0	Any
Stage 3	III	3	0	0	Any
Stage 4	IV	4	0	0	Any
Stage 4	IV	Any	1	0	Any
Stage 4	IV	Any	Any	1	Any

SUPPLEMENTARY TABLE 'A' – EXPOSURE VARIABLES EXPLAINED

		Name	Description	How operationalised
Patient level		Patient age	Patient age from cancer registry, 2012	Grouped into age bands. Adults only.
		Patient sex	Patient sex from cancer registry, 2012	
		Patient ethnicity	Patient ethnicity from cancer registry, 2012	Categorised into 'white' and 'non-white'. Due to small numbers of non-white ethnicity unable to sub-group further.
		Patient level deprivation	Patient level deprivation from cancer registry, 2012. Derived from patient postcode using income domain of index of multiple deprivation (IMD) 2010.	Quintiles
GP level	Demographic factors	Training practice	Whether practice is a training practice or not, i.e. whether it has GP registrars. From GP workforce survey 2012.	Binary
		GPs 50 years and over	Proportion of GPs working at practice aged 50 years and over. From GP workforce survey 2012.	Divided into 'some', 'none' or 'all', as per previous studies. (Bottle *et al*, 2012)
		GPs female	Sex of GPs working at practice. From GP workforce survey 2012.	Divided into 'some', 'none' or 'all', as per previous studies. (Bottle *et al*, 2012)
		GPs primary UK qualification	Whether GPs primary medical qualification was from the UK. From GP workforce survey 2012.	Divided into 'some', 'none' or 'all', as per previous studies. (Bottle *et al*, 2012)
		GP income deprivation	The index of multiple deprivation is derived from 7 domains; income, employment, health & disability, education skills & training, barriers to housing & services, crime and living environment. The GP practice income IMD is estimated by taking a weighted average of the income IMD scores of each LSOA in which a given practice has registrations. The weights are % of the practice's registrations in each LSOA.<br>From NHS Health and Social Care Centre, 2011.	Quintiles
		Rurality	Rurality of the GP practice is based on population density of the practice postcode, from 2001 census. Data from Health and Social Care Information Centre, 2011.	Pre-determined categories.
		Number of patients per GP	Calculated as list size divided by GP full time equivalent, to give average number of patients per GP as each practice. GP practice list size from QOF 2011/12. Full time equivalent GP practitioners from GP workforce survey 2012.	Quintiles
		Total QOF points	Total points from Quality Outcome Framework, 2011/12. Maximum achievable 1,000.	Divided into groups based on spread of data.
	General	Able to book	Percentage of patients responding 'yes' to question 'Were you able to get an	Divided into categories based on spread of

	factors	appointment	appointment see or speak to someone?' within GP Survey 2011/12.<br><br>Weighted responses have been used as these try to remove any bias introduced by response bias (adjusts the data to account for potential differences between the demographic profile of all eligible patients in a practice and the patients who actually completed the questionnaire). In 2011/12, 1 million people responded to the question 'were you able to get an appointment to see or speak to someone'.	data. Division into categories makes easier for interpretation than division into tertiles.
		Able to see preferred GP	Percentage of patients responding 'always', 'almost always' or 'a lot of the time' to question 'Were you able to see your preferred doctor?' within 2010/11 GP Survey (of those that said they had a preferred doctor).<br><br>Weighted responses have been used as these try to remove any bias introduced by response bias (adjusts the data to account for potential differences between the demographic profile of all eligible patients in a practice and the patients who actually completed the questionnaire). In 2010/11, 1.17million (% of 1.93 who answered question) had a preferred doctor with 1.16 million of these (99%) answering the question 'how often do you see your preferred doctor'.	Divided into categories based on spread of data and easier for interpretation than division into tertiles.<br>Data from 2010/11 used instead of 2011/12 as more complete. Strong correlation between 2011/12 and 2010/11 data. Due to the changes to the questionnaire design and survey frequency, as well as the change to the weighting methodology results from 2011/12 onwards cannot be compared with previous years, therefore 2010/11 data was not input for missing data of 2011/12.
	Cancer specific factors	Two week wait referral rate	Two week wait referrals 2011/12. Number per 100,000 population. From National Cancer Intelligence Network Practice Profiles (NCIN), 2012.	Divided into quintiles due to spread of data.
		Two week wait conversion	Two week wait conversion 2011/12. Percentage of all two week wait referrals with cancer. From NCIN Practice Profiles, 2012.	Quintiles
		Two week wait detection	Number of new cancers treated, percentage of which are two week wait 2011/12. From National Cancer Intelligence Network Practice Profiles, 2012.	Quintiles
	Other factors	Average colonoscopy, sigmoidoscopy and endoscopy rate	Average of in-patient or day case colonoscopy, sigmoidoscopy and upper gastrointestinal endoscopy. Number per 100,000 population. From National Cancer Intelligence Network Practice Profiles, 2012.	Divided into tertiles due to spread of data and as per previous study. (Shawihdi *et al*, 2012)
		Emergency presentations	Number of persons diagnosed via an emergency route, as defined by the Routes to Diagnosis project methodology.[i] Percentage of presentations. From National Cancer Intelligence Network Practice Profiles, 2012.	Quintiles

i. Elliss-Brookes L, McPhail S, Ives A, Greenslade M, Shelton J, Hiom S, et al. Routes to diagnosis for cancer – determining the patient journey using multiple data sets. Br J Cancer. 2012;107(8):1220-6.

SUPPLEMENTARY FIGURE 'A' – CONCEPTUAL MODEL

We felt that the GP characteristics included in the study measured certain aspects of primary care associated with the stage of cancer at diagnosis. These are shown in the conceptual model below, and appeared to be likely to be related with one another along the causal pathway. A number of these will impact on primary care delay, whilst some will impact on patient delay.

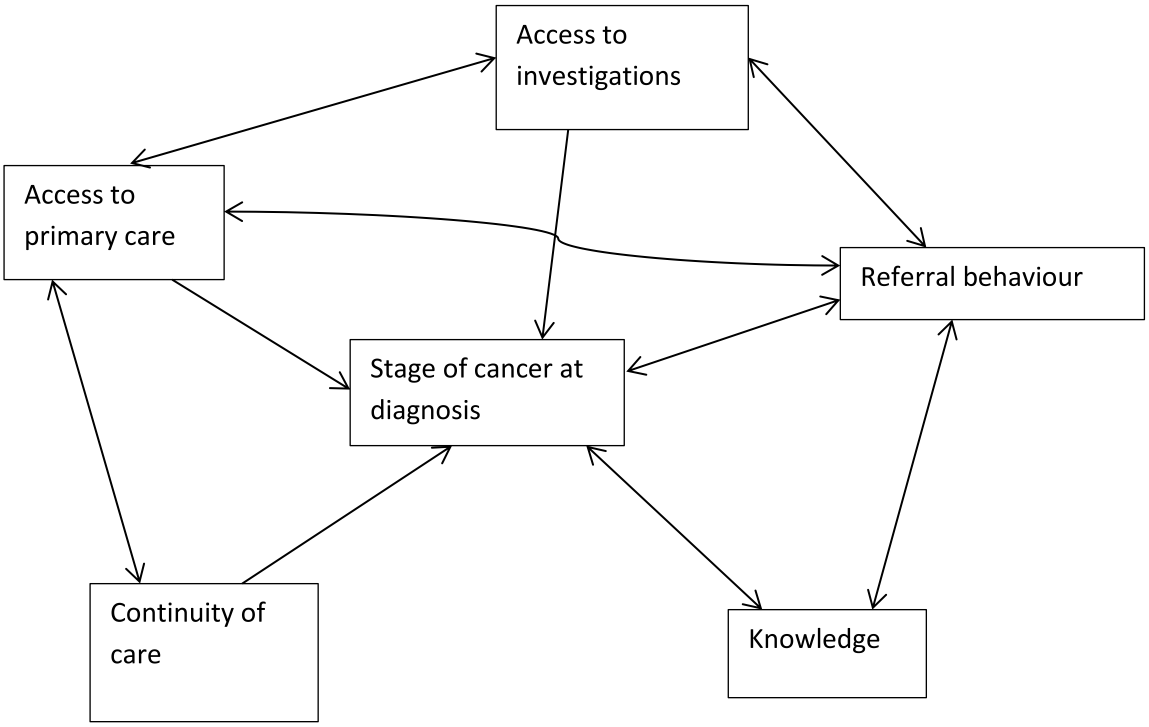

SUPPLEMENTARY TABLE 'B' – NUMBER AND PERCENTAGE OF TUMOURS OF EACH CANCER TYPE AND STAGE

	Exposure variables	Female breast cancer (n=34,119)		Prostate cancer (n=27,880)		Colorectal cancer (n=27,079)		Lung cancer (n=28,479)	
		Stage 1&2 (n=28,453)	Stage 3&4 (n=5,666)	Stage 1&2 (n=17,124)	Stage 1&2 (n=17,124)	Stage 3&4 (n=10,756)	Stage 3&4 (n=14,793)	Stage 1&2 (n=6,959)	Stage 3&4 (n=21,520)
Patient level	**Age**								
	15-44 years	2,652 (9.3%)	663 (11.7)	47 (0.3)	13 (0.1)	332 (2.7)	561 (3.8)	86 (1.2)	221 (1.0)
	45-64 years	13,388 (47.1)	2,300 (40.6)	4,845 (28.3)	2,346 (21.8)	2,905 (23.5)	4,096 (27.8)	1,437 (20.6)	5,340 (24.8)
	65+ years	12,413 (43.6)	2,703 (47.7)	12,232 (71.4)	8,397 (78.1)	9,103 (73.8)	10,082 (68.4)	5,436 (78.1)	15,959 (74.2)
	**Sex**								
	Male	--------- ------	--------- ------	17,124 (100)	10,756 (100)	7,055 (57.2)	8,406 (57.0)	3,606 (51.8)	12,054 (56.0)
	Female	28,453 (100)	5,666 (100)	--------- ------	--------- ------	5,285 (42.8)	6,333 (43.0)	3,353 (48.2)	9,466 (44.0)
	**Ethnicity**								
	White	17,331 (60.9)	3,464 (61.1)	8,228 (48.0)	5,773 (53.7)	7,757 (62.9)	9,752 (65.9)	4,729 (68.0)	14,514 (67.4)
	Non-white	786 (2.8)	230 (4.1)	342 (2.0)	186 (1.7)	229 (1.9)	380 (2.6)	127 (1.8)	375 (1.7)
	Missing	10,336 (36.3)	1,972 (34.8)	8,554 (50.0)	4,797 (44.6)	4,354 (35.3)	4,661 (31.5)	2,103 (30.2)	6,631 (30.8)
	**Deprivation**								
	Q1 (least deprived)	6,271 (22.0)	1,167 (20.6)	4,147 (24.2)	2,347 (21.8)	2,681 (21.7)	3,169 (21.5)	956 (13.7)	3,008 (14.0)
	Q2	6,602 (23.2)	1,172 (20.7)	4,237 (24.7)	2,625 (24.4)	2,772 (22.5)	3,222 (21.9)	1,294 (18.6)	3,973 (18.5)
	Q3	6,276 (22.1)	1,169 (20.6)	3,706 (21.6)	2,356 (21.9)	2,702 (21.9)	3,153 (21.4)	1,384 (19.9)	4,433 (20.6)
	Q4	5,200 (18.3)	1,159 (20.5)	2,866 (16.7)	1,930 (17.9)	2,317 (18.8)	2,853 (19.4)	1,566 (22.5)	4,855 (22.6)
	Q5 (most deprived)	4,104 (14.4)	999 (17.6)	2,168 (12.7)	1,498 (13.9)	1,868 (15.1)	2,342 (15.9)	1,759 (25.3)	5,251 (24.4)
GP practice level - demographics	**Number of patients per GP**								
	Q1 (lowest)	5,700 (20.0)	1,147 (20.2)	3,417 (20.0)	2,241 (20.8)	2,542 (20.6)	2,938 (19.9)	1,443 (20.7)	4,236 (19.7)
	Q2	5,606 (19.7)	1,120 (19.8)	3,338 (19.5)	2,084 (19.4)	2,422 (19.6)	2,915 (19.8)	1,422 (20.4)	4,298 (20.0)
	Q3	5,777 (20.3)	1,098 (19.4)	3,408 (19.9)	2,153 (20.0)	2,383 (19.3)	2,878 (19.5)	1,373 (19.7)	4,170 (19.4)
	Q4	5,525 (19.4)	1,137 (20.1)	3,394 (19.8)	2,156 (20.0)	2,449 (19.8)	3,021 (20.5)	1,345 (19.3)	4,323 (20.1)
	Q5 (highest)	5,845 (20.5)	1,164 (20.5)	3,567 (20.8)	2,122 (19.7)	2,544 (20.6)	2,987 (20.3)	1,376 (19.8)	4,493 (20.9)
	**Training practice**								
	No	15,802 (55.5)	3,241 (57.2)	9,752 (56.9)	6,039 (56.1)	6,958 (56.4)	8,345 (56.6)	2,915 (41.9)	8,850 (41.1)
	Yes	12,651 (44.5)	2,425 (42.8)	7,372 (43.1)	4,717 (43.9)	5,382 (43.6)	6,394 (43.4)	4,044 (58.1)	12,670 (58.9)
	**GPs aged 50 and over**								
	Some	24,713 (86.9)	4,876 (86.1)	14,766 (86.2)	9,337 (86.8)	10,631 (86.2)	12,721 (86.3)	5,899 (84.8)	18,337 (85.2)
	None	2,148 (7.5)	442 (7.8)	1,309 (7.6)	812 (7.5)	996 (8.1)	1,135 (7.7)	657 (9.4)	1,785 (8.3)
	All	1,592 (5.6)	348 (6.1)	1,049 (6.1)	607 (5.6)	713 (5.8)	883 (6.0)	403 (5.8)	1,398 (6.5)
	**GPs female**								
	Some	25,939 (91.2)	5,136 (90.6)	15,470 (90.3)	9,744 (90.6)	11,179 (90.6)	13,394 (90.9)	6,253 (89.9)	19,305 (89.7)
	None	2,069 (7.3)	408 (7.2)	1,381 (8.1)	853 (7.9)	958 (7.8)	1,129 (7.7)	563 (8.1)	1,864 (8.7)
	All	445 (1.6)	122 (2.2)	273 (1.6)	159 (1.5)	203 (1.6)	216 (1.5)	143 (2.1)	351 (1.6)
	**GPs qualified in UK**								
	Some	16,964 (59.6)	3,391 (59.8)	10,043 (58.6)	6,260 (58.2)	7,210 (58.4)	8,692 (59.0)	4,158 (59.7)	12,938 (60.1)
	None	1,543 (5.4)	342 (6.0)	1,003 (5.9)	568 (5.3)	704 (5.7)	846 (5.7)	453 (6.5)	1,367 (6.4)
	All	9,946 (35.0)	1,933 (34.1)	6,078 (35.5)	3,928 (36.5)	4,426 (35.9)	5,201 (35.3)	2,348 (33.7)	7,215 (33.5)
	**GP level deprivation**								
	Q1 (least deprived)	6,448 (22.7)	1,197 (21.1)	4,202 (24.5)	2,401 (22.3)	2,701 (21.9)	3,261 (22.1)	1,077 (15.5)	3,551 (16.5)
	Q2	6,719 (23.6)	1,286 (22.7)	4,126 (24.1)	2,559 (23.8)	2,853 (23.1)	3,279 (22.2)	1,420 (20.4)	4,244 (19.7)
	Q3	6,206 (21.8)	1,161 (20.5)	3,614 (21.1)	2,364 (22.0)	2,690 (21.8)	3,114 (21.1)	1,385 (19.9)	4,492 (20.9)
	Q4	5,311 (18.7)	1,074 (19.0)	2,996 (17.5)	1,982 (18.4)	2,349 (19.0)	2,897 (19.7)	1,539 (22.1)	4,831 (22.4)
	Q5 (most deprived)	3,769 (13.2)	948 (16.7)	2,186 (12.8)	1,450 (13.5)	1,747 (14.2)	2,188 (14.8)	1,538 (22.1)	4,402 (20.5)
	**GP rurality**								
	Urban	22,789 (80.1)	4,696 (82.9)	13,405 (78.3)	8,575 (79.7)	9,975 (80.8)	11,974 (81.2)	5,909 (84.9)	18,259 (84.8)
	Town	4,583 (16.1)	786 (13.9)	2,938 (17.2)	1,723 (16.0)	1,904 (15.4)	2,219 (15.1)	871 (12.5)	2,686 (12.5)
	Village	1,081 (3.8)	184 (3.2)	781 (4.6)	458 (4.3)	461 (3.7)	546 (3.7)	179 (2.6)	575 (2.7)

GP practice level - general performance	**Able to book appointment**																
	90% and over	12,456	(43.8)	2,350	(41.5)	7,893	(46.1)	4,890	(45.5)	5,453	(44.2)	6,474	(43.9)	2,764	(39.7)	8,729	(40.6)
	80-90%	13,671	(48.0)	2,772	(48.9)	7,804	(45.6)	5,028	(46.7)	5,873	(47.6)	7,006	(47.5)	3,535	(50.8)	10,761	(50.0)
	<80%	2,326	(8.2)	544	(9.6)	1,427	(8.3)	838	(7.8)	1,014	(8.2)	1,259	(8.5)	660	(9.5)	2,030	(9.4)
	**Able to see preferred GP**																
	80% and over	7,553	(26.5)	1,423	(25.1)	4,890	(28.6)	3,077	(28.6)	3,389	(27.5)	4,075	(27.6)	1,715	(24.6)	5,614	(26.1)
	60-80%	14,229	(50.0)	2,824	(49.8)	8,335	(48.7)	5,298	(49.3)	6,171	(50.0)	7,255	(49.2)	3,464	(49.8)	10,508	(48.8)
	<60%	6,671	(23.4)	1,419	(25.0)	3,899	(22.8)	2,381	(22.1)	2,780	(22.5)	3,409	(23.1)	1,780	(25.6)	5,398	(25.1)
	**Total QOF points**																
	990 to 1000 points	13,836	(48.6)	2,690	(47.5)	8,466	(49.4)	5,189	(48.2)	6,029	(48.9)	7,184	(48.7)	3,259	(46.8)	10,270	(47.7)
	980 to 989 points	6,279	(22.1)	1,201	(21.2)	3,636	(21.2)	2,364	(22.0)	2,670	(21.6)	3,197	(21.7)	1,496	(21.5)	4,518	(21.0)
	960 to 979 points	4,771	(16.8)	1,011	(17.8)	2,861	(16.7)	1,841	(17.1)	2,062	(16.7)	2,515	(17.1)	1,250	(18.0)	3,859	(17.9)
	<960 points	3,567	(12.5)	764	(13.5)	2,161	(12.6)	1,362	(12.7)	1,579	(12.8)	1,843	(12.5)	954	(13.7)	2,873	(13.4)
GP practice level- specific cancer activity	**Two week wait referral rate**																
	Q1 (lowest)	5,715	(20.1)	1,266	(22.3)	3,424	(20.0)	2,081	(19.3)	2,457	(19.9)	2,984	(20.2)	1,354	(19.5)	4,640	(21.6)
	Q2	5,622	(19.8)	1,096	(19.3)	3,408	(19.9)	2,077	(19.3)	2,464	(20.0)	3,035	(20.6)	1,428	(20.5)	4,464	(20.7)
	Q3	5,561	(19.5)	1,176	(20.8)	3,393	(19.8)	2,209	(20.5)	2,462	(20.0)	2,919	(19.8)	1,383	(19.9)	4,181	(19.4)
	Q4	5,876	(20.7)	1,057	(18.7)	3,431	(20.0)	2,215	(20.6)	2,504	(20.3)	2,959	(20.1)	1,345	(19.3)	4,104	(19.1)
	Q5 (highest)	5,679	(20.0)	1,071	(18.9)	3,468	(20.3)	2,174	(20.2)	2,453	(19.9)	2,842	(19.3)	1,449	(20.8)	4,131	(19.2)
	**Two week wait conversion**																
	Q1 (lowest)	5,850	(20.6)	1,251	(22.1)	3,391	(19.8)	2,029	(18.9)	2,469	(20.0)	3,020	(20.5)	1,619	(23.3)	4,503	(20.9)
	Q2	5,627	(19.8)	1,119	(19.7)	3,127	(18.3)	2,047	(19.0)	2,315	(18.8)	2,919	(19.8)	1,411	(20.3)	4,211	(19.6)
	Q3	5,798	(20.4)	1,096	(19.3)	3,475	(20.3)	2,177	(20.2)	2,518	(20.4)	2,786	(18.9)	1,400	(20.1)	4,148	(19.3)
	Q4	5,675	(19.9)	1,107	(19.5)	3,555	(20.8)	2,210	(20.5)	2,643	(21.4)	3,007	(20.4)	1,262	(18.1)	4,258	(19.8)
	Q5 (highest)	5,503	(19.3)	1,093	(19.3)	3,576	(20.9)	2,293	(21.3)	2,395	(19.4)	3,007	(20.4)	1,267	(18.2)	4,400	(20.4)
	**Two week wait detection**																
	Q1 (lowest)	5,297	(18.6)	1,160	(20.5)	3,458	(20.2)	2,077	(19.3)	2,546	(20.6)	2,980	(20.2)	1,436	(20.6)	4,641	(21.6)
	Q2	5,418	(19.0)	1,144	(20.2)	3,466	(20.2)	2,126	(19.8)	2,420	(19.6)	3,006	(20.4)	1,432	(20.6)	4,356	(20.2)
	Q3	6,411	(22.5)	1,255	(22.1)	3,891	(22.7)	2,536	(23.6)	2,793	(22.6)	3,359	(22.8)	1,571	(22.6)	4,816	(22.4)
	Q4	5,032	(17.7)	911	(16.1)	2,756	(16.1)	1,860	(17.3)	2,198	(17.8)	2,450	(16.6)	1,136	(16.3)	3,534	(16.4)
	Q5 (highest)	6,295	(22.1)	1,196	(21.1)	3,553	(20.7)	2,157	(20.1)	2,383	(19.3)	2,944	(20.0)	1,384	(19.9)	4,173	(19.4)
GP practice level - other	**Average colonoscopy, sigmoidoscopy and upper GI endoscopy**																
	T1 (lowest)	9,789	(34.4)	1,962	(34.6)	5,944	(34.7)	3,489	(32.4)	3,958	(32.1)	4,905	(33.3)	2,136	(30.7)	6,836	(31.8)
	T2	9,335	(32.8)	1,761	(31.1)	5,595	(32.7)	3,624	(33.7)	4,026	(32.6)	4,932	(33.5)	2,261	(32.5)	6,801	(31.6)
	T3 (highest)	9,329	(32.8)	1,943	(34.3)	5,585	(32.6)	3,643	(33.9)	4,356	(35.3)	4,902	(33.3)	2,562	(36.8)	7,883	(36.6)
	**Emergency admissions**																
	Q1 (lowest)	5,747	(20.2)	1,231	(21.7)	3,514	(20.5)	2,086	(19.4)	2,353	(19.1)	2,866	(19.4)	1,341	(19.3)	3,989	(18.5)
	Q2	5,891	(20.7)	1,104	(19.5)	3,486	(20.4)	2,181	(20.3)	2,474	(20.0)	2,992	(20.3)	1,344	(19.3)	4,084	(19.0)
	Q3	5,625	(19.8)	1,177	(20.8)	3,438	(20.1)	2,193	(20.4)	2,474	(20.0)	2,933	(19.9)	1,338	(19.2)	4,204	(19.5)
	Q4	5,731	(20.1)	1,144	(20.2)	3,400	(19.9)	2,167	(20.1)	2,509	(20.3)	2,972	(20.2)	1,454	(20.9)	4,506	(20.9)
	Q5 (highest)	5,459	(19.2)	1,010	(17.8)	3,286	(19.2)	2,129	(19.8)	2,530	(20.5)	2,976	(20.2)	1,482	(21.3)	4,737	(22.0)
